# The impact of antiretroviral therapy on symptom burden among HIV outpatients with low CD4 count in rural Uganda: nested longitudinal cohort study

**DOI:** 10.1186/s12904-017-0215-y

**Published:** 2017-07-13

**Authors:** Katie Wakeham, Richard Harding, Jonathan Levin, Rosalind Parkes-Ratanshi, Anatoli Kamali, David G Lalloo

**Affiliations:** 1 MRC/UVRI Uganda Research Unit on AIDS, Entebbe, Uganda; 2 Liverpool School of Tropical Medicine, Liverpool, UK; 3 Sussex Cancer Centre, Brighton and Sussex University Hospital, Eastern Road, Brighton, BN2 5DA UK; 4 Department of Palliative Care, Policy & Rehabilitation, King’s College London, Cicely Saunders Institute, London, UK; 5 School of Public Health, Faculty of Health Sciences, University of Witwatersrand, Johannesburg, South Africa

**Keywords:** Symptom burden, Symptom control, Palliative care, HIV, Africa, ART, Pain

## Abstract

**Background:**

Individuals with HIV have a high prevalence of physical and psychological symptoms throughout their disease course. Despite the clinical and public health implications of unresolved pain and symptoms, little is known about the effect of anti-retroviral therapy (ART) on these outcomes. This study aimed to assess the impact on symptom burden for the year after ART initiation in individuals with a CD4 count <200 cells/uL in Uganda.

**Methods:**

HIV-infected, ART-naıve adults referred from voluntary testing and counseling services in rural Uganda for enrollment into a randomized controlled trial to test fluconazole as primary prophylaxis against cryptococcal disease were invited to complete the Memorial Symptom Assessment Scale-Short Form (MSAS-SF) prior to commencing ART and at two subsequent follow up visits. This tool measures self-reported 7-day period prevalence and associated burden of physical and psychological symptoms. Changes in the total number of symptoms and distress indices with time on ART and trial arm were investigated through fitting Linear Mixed Models for repeated measures.

**Results:**

During the first year of ART initiation the prevalence of most individual symptoms remained constant. The notable exceptions which improved after commencing ART are as follow; prevalence of pain (prevalence changed from 79% to 60%), weight loss (67% to 31%), lack of appetite (46% to 28%), feeling sad (52% to 25%) and difficulty sleeping (35% to 23%). The total number of symptoms and distress indices reduced after treatment commenced. Of concern was that half or more study participants remained with symptoms of pain (60%), itching (57%), skin changes (53%) and numbness in hands and feet (52%) after starting ART. Sixteen symptoms remained with a burden of 25% or more.

**Conclusion:**

Despite the beneficial effect of ART on reducing symptoms, some patients continue to experience a high symptom burden. It is essential that HIV services in sub-Saharan Africa integrate management of symptoms into their programmes.

**Trial registration:**

CRYPTOPRO [ISRCTN 76481529], November 2004.

**Electronic supplementary material:**

The online version of this article (doi:10.1186/s12904-017-0215-y) contains supplementary material, which is available to authorized users.

## Background

An estimated 36.7 million individuals in Sub-Saharan Africa are infected with HIV [1]. The rapid roll-out of anti-retroviral therapy (ART) has led to a treatment coverage of approximately 50% [1]. ART suppresses viral replication, restores immune function and has led to significant reductions in morbidity and improved survival. Where treatment is available, HIV infection is transitioning into a chronic disease. A high prevalence of troublesome symptoms is reported at all stages of HIV infection and persists during treatment [2–5]; if identified, these symptoms can be successfully controlled [6]. Growing evidence suggests that unpleasant symptoms are associated with poor treatment adherence [7], unprotected sexual intercourse [8], HIV viral load rebound [9], and reduced quality of life [10] potentially influencing HIV transmission [7, 11]. Furthermore, HIV positive people receiving ART report a higher prevalence of mental health illness [5] and perceived stigma than the general population [12]. Therefore, managing physical and psychological symptoms through palliation becomes a major challenge in HIV care.

We have previously published evidence of a high prevalence and burden of physical and psychological symptoms among individuals in Uganda who were eligible to begin receiving ART but had not yet started ART [4]. Studies that have investigated longitudinal changes in global quality of life of people taking ART in sub-Saharan Africa suggest improvements in some measured outcomes [13–15]. Individuals with HIV may have multiple physical and psychological symptoms and a detailed assessment and management of illness burden is a vital part of quality of life assessment. However, to date there has been a paucity of longitudinal reports describing detailed clinical symptomatology as an outcome for HIV-infected individuals in sub-Saharan Africa accessing treatment. This study aimed to measure longitudinal symptom burden from before commencing ART and during the first year of treatment in a population of adults with a CD4 count <200 cells /uL in Uganda.

## Methods

### Setting

The Symptom Burden Longitudinal Cohort Study was conducted in a rural district in South Western Uganda. It was conducted within a double-blind randomised placebo controlled trial which determined that primary prophylaxis with fluconazole reduced morbidity and mortality from invasive cryptococcal disease in HIV infected Ugandan adults [16]. ART-naive adults were referred to the CRYPTOPRO study clinic following voluntary testing and counselling at one of the five local HIV care and treatment organisations.

### Study population

In line with the selection criteria of the main study, study subjects comprised consenting ART-naive adults, with laboratory confirmation of HIV, CD4 count <200 cells/uL, and a negative cryptococcal antigen test. Exclusion criteria included pregnancy and liver function tests greater than three times the upper limit of normal [16]. Participants received either 200 mg of fluconazole or an identical placebo (Cipla, India) three times per week from the baseline enrolment visit until the end of the trial (minimum 12 weeks) or until their CD4 count reached 200 cells/μl.

### Antiretroviral therapy

Local ART providers managed initiating ART independently of the CRYPTOPRO trial team and chose the drug combination and monitored antiretroviral regimens (two nucleoside reverse transcriptase inhibitors and one non-nucleoside reverse transcriptase inhibitor). Patients were offered routine co-trimoxazole prophylaxis (960 mg three times a week) according to standard Ugandan guidelines.

### Symptom burden procedure and tools

All ART-naïve participants enrolling in CRYPTOPRO on one randomly chosen day per week between 2007 and 2008 were invited to complete the Memorial Symptom Assessment Scale-Short Form (MSAS-SF) [17] with a trained counsellor. The MSAS-SF was translated into local languages, back-translated, and administered to clients in the language of their choice (available as Additional file 1) . The MSAS-SF was then repeated at either one or two follow-up visits after commencing ART over a one-year period. MSAS-SF is a patient-rated symptom measurement tool, which records the seven-day period prevalence and associated burden of 28 physical and four psychological symptoms. For each symptom the respondent indicated whether or not they have experienced it in the previous 7 days. For those symptoms experienced, each is then scored according to perceived burden. A burden score is allocated to each prevalent physical symptom depending on the level of discomfort reported (not at all = 0.8, a little = 1.6, somewhat = 2.4, quite a bit = 3.2, very much = 4). Similarly, a score is allocated to each prevalent psychological symptom depending on the frequency (rarely = 1, occasionally = 2, frequently = 3, almost constantly = 4). For any symptom (either physical or psychological) that is not experienced, a burden score of zero is allocated. Each MSAS-SF subscale is then calculated from the mean burden score for a pre-defined subset of the items. The Global Distress Index (GDI) is based on 10 parameters; six physical symptoms (pain, lack of energy, lack of appetite, dry mouth, drowsiness and constipation) and four psychological factors (feeling sad, worried, irritated and nervous). The Physical Symptom Distress Score (PHYS) is based on 12 physical symptoms (pain, lack of energy, lack of appetite, feeling drowsy, constipation, dry mouth, nausea, vomiting, change in taste, weight loss, feeling bloated and dizziness). The Psychological Symptom Distress Score (PSYCH) is based on burden of six psychological symptoms (feeling sad, worried, nervous, difficulty sleeping, feeling irritable and difficulty concentrating). Significant symptom distress is suggested by a MSAS-SF subscales scores greater than one. A pilot study of MSAS-SF in Uganda (personal communication, Dr. K. Frame) identified a further nine prevalent physical symptoms (hunger, difficulty moving, difficulty walking, muscle aches, poor vision, poor hearing, sore lumps on private parts, discharge from private parts and bad smell/body odour,) and these were added to our questionnaire, as described previously [4].

### Statistical analysis

Data from hand-annotated symptom burden questionnaire were entered into a database created in MS-ACCESS and patient data was extracted from the main CRYPTOPRO study database. The prevalence of each symptom was tabulated to allow comparison between the pre- and post-ART visits. Individual physical symptom burden reported as “quite a bit” or “very much” was regarded as experiencing “high distress”. Similarly, individuals who reported a psychological symptom causing distress “frequently” or “constantly” were regarded as experiencing “high frequency” distress. The three subscale symptom distress indices (GDI, PHYS and PSYCH) were calculated from the mean burden scores. Changes in subscale symptom distress indices with time on ART was assessed by fitting Linear Mixed Models (LMMs) for repeated measurements [18, 19] to investigate whether, and if so, how, the total symptom score, the global distress index, the physical symptom score and the psychological symptom score changed with time on ART. Scores recorded before initiation of ART were assumed to be at time zero, for other observations time was number of days on ART. Regression analyses carried out on the scales at baseline suggested the use of a square root transformation of the outcome in order to satisfy the assumption of homogeneity of variance. Thus the square-root of each scale was used as the outcome variable, which also ensured that predicted values from the model would always be positive after back-transformation to the original scale. In addition, the models investigated whether there was evidence of non-linearity of the effect of time on ART; the models were fitted using Maximum Likelihood Estimation (MLE) rather than the more conventional Residual Maximum Likelihood Estimation (REML) in order to assess extra fixed effect terms in the model. A quadratic term in days post-ART was added if it significantly increased the model deviance (minus twice the log-likelihood) at the 5% level; the square root of days since ART was used instead of a quadratic model if it resulted in a lower residual deviance than the quadratic model. The number of days post-ART was scaled by dividing by 100 to give greater numerical stability.

Further linear mixed models were fitted to investigate whether the total number of symptoms and the symptom scores were associated with either the ART regimen or the CRYPTOPRO treatment arm (fluconazole vs. placebo), adjusting for CD4 cell count and length of time on ART.

All analyses were carried out using Stata release 11.2 (College Station, TX).

### Ethics

This study received ethical approval from the Uganda Virus Research Institute and Uganda National Council for Science and Technology.

## Results

### Study participant characteristics

From the cohort the MSAS-SF was completed by 97 participants at study baseline before and after initiating ART (Table 1). The median length of time on ART was 300 days (IQR 140–377). Individuals completed two or three data points after initiating ART.

**Table 1:** Study participants characteristics (*n* = 97)

Factor		*n* (%)
Sex	Male	30 (31%)
	Female	67 (69%)
Age	Mean (s.d.)	36 (8)
WHO HIV stage	1	3 (3%)
	2	34 (35%)
	3	54 (56%)
	4	6 (6%)
CD4 cells (cells/ul)	<50	22 (23%)
	50–99	19 (19%)
	100–149	24 (25%)
	150–199	32 (33%)
Main study treatment arm	Median (IQR)	120 (58–163)
	Fluconazole	47 (48%)
	Placebo	50 (52%)
Anti-retroviral treatment backbone (missing = 2)	AZT/3TC	77 (81%)
	D4T/3TC	16 (17%)
	Truvada	2 (2%)
Non-nucleoside reverse-transcriptase inhibitors (missing = 1)	Nevirapine	70 (73%)
	Efavirenz	26 (27%)
Died during study	No	88 (91%)
	Yes	9 (9%)

*AZT* zidovudine; *3TC* lamivudine; d4T Stavudine; Truvada emtricitabine and tenofovir disoproxil fumarate

### Seven-day period prevalence of symptoms and symptom distress indices

The prevalence of pain, weight loss, lack of appetite, feeling sad, difficulty sleeping and walking, problems urinating, irritability, feeling nervous and mouth sores reduced in frequency during the early phases of taking ART (Table 2). In cases where individuals had more than one follow-up visit while on ART, the symptom burden at the longest time on ART has been used. The remaining 31 symptoms remained at a similar burden with 16 persistent symptoms having a frequency of over 25%. While the prevalence of pain reduced from 79 to 60% on commencing ART, the burden remains high and of those with pain, 25% reported high distress associated with this symptom. Of concern is that half or more study participants remained with symptoms of itching (%), skin changes (%) and numbness (%) after starting ART. A high prevalence of distress at follow-up was reported for sexual activity (36%), hunger (34%), changes to skin (27%), pain (25%), numbness and tingling (22%), feeling sad (21%) and worry (21%) (Table 2). New symptoms after initiating ART were particularly prevalent for hunger (26%), problems with sexual activity (25%) and cough (20%) (Table 2).

**Table 2:** Frequency of symptoms in past week prior to starting ART and frequency, distress and prevalence of new symptoms after starting ART for 97 study participants

Symptom	Burden of symptoms prior to starting ART [n (%)]	Burden of symptoms after starting ART [n (%)]	Prevalence of high distress in participants with symptom after starting ART [n [%)]
Pain	77 (79%)	58 (60%)	34 (25%)
Itching	69 (69%)	55 (57%)	30(31%)
Changes in skin	49 (51%)	51 (53%)	26 (27%)
Numbness/tingling in the hands/feet	52 (54%)	50 (52%)	21 (22%)
Hunger	47 (43%)	47 (43%)	33 (34%)
Cough	44 (45%)	45 (46%)	14(14%)
Worrying	58 (60%)	44 (45%)	20(21%)
Lack of energy	51 (52%)	42 (43%)	17(18%)
Problems with sexual interest/activity	37 (33%)	41 (42%)	35 (36%)
Feeling drowsy/tired	53 (55%)	40 (41%)	11 (11%)
Weight loss	65 (67%)	30 (31%)	11 (11%)
Lack of appetite	45 (46%)	27 (23%)	16(16%)
Sores or lumps on private parts	22 (29%)	27 (23%)	11 (11%)
Dizziness	38 (39%)	26(27%)	12(12%)
Changes in the way food tastes	30 (31%)	24 (25%)	9 (9%)
Feeling sad	50 (52%)	24 (25%)	20(21%)
Difficulty sleeping	34 (35%)	22 (23%)	12(12%)
Dry mouth	32 (33%)	20 (21%)	5 (5%)
Nausea	20 (21%)	20 (21%)	8 (8%)
Difficulty walking	35 (36%)	19 (20%)	7(7%)
I do not like myself	28 (29%)	18 (19%)	13(13%)
Muscle aches	27 (28%)	18 (19%)	9 (9%)
Difficulty seeing well - poor vision	18 (19%)	17 (18%)	6 (6%)
Constipation	16 (16%)	15 (15%)	7(7%)
Problems urinating	18 (19%)	14 (14%)	5 (5%)
Sweats	29 (30%)	14 (14%)	4 (4%)
Diarrhea	16 (16%)	13 (13%)	7(7%)
Feeling bloated	17 (18%)	13 (13%)	4 (4%)
Difficulty swallowing	I5 (16%)	12 (12%)	11 (11%)
Feeling irritable	26 (27%)	11(11%)	2 (2%)
Difficulty concentrating	7 (7%)	10 (10%)	3 (3%)
Difficulty moving	14 (14%)	10 (10%)	2 (2%)
Shortness of breath	16 (17%)	10 (10%)	4 (4%)
Bad smell/odour from body	8(8%)	8(8%)	2 (2%)
Feeling nervous	27 (28%)	8(8%)	4 (4%)
Difficulty hearing well - poor hearing	6 (6%)	7(7%)	2 (2%)
Hair loss	11 (11%)	7(7%)	3 (3%)
Swelling of arms or legs	9 (9%)	7(7%)	3 (3%)
Discharge from private parts	15 (16%)	6(6%)	4 (4%)
Vomiting	8 (8%)	5(5%)	1 (1%)
Mouth sores	19 (20%)	3(3%)	3 (3%)

The proportions of participants experiencing numbness or tingling after starting ART were similar for those on an AZT based regimen (51%) and those on a d4T-based regimen (56%). The prevalence of symptoms that could potentially be altered by the trial drug fluconazole, namely pain, weight loss, lack of appetite, sore or discharging private parts, difficult swallowing and mouth sores did not differ between those on the active drug and the placebo in this study.

### Changes in subscale symptom distress indices with time on ART

**Fig. 1:**
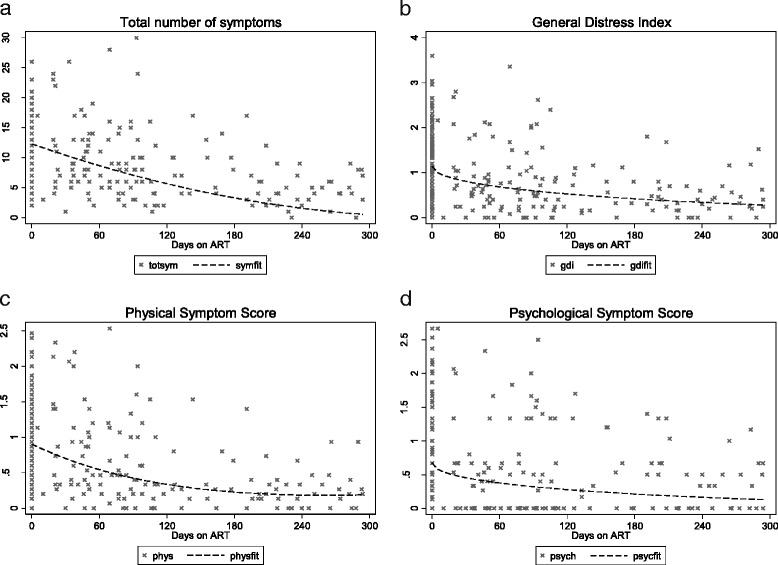
Observed values and fitted curves using a linear mixed model with study participants regarded as having random effects for total number of symptoms (panel **a**), Global Distress Index (panel **b**), Physical Symptom Distress Score (panel **c**) and Psychological Symptom Distress Score (panel **d**)

The mean total number of symptoms decreased after commencing ART from 12.8 (11.7–13.9) to 7.8 (6.8–8.9) (p = <0.0001) (Table 3). The summary scores for GDI, PHYS and PSYCH all approximately halved after starting ART (Table 3). The change in the total number of symptoms and the three indices with time on ART were investigated through fitting LMMs for repeated measures; the number of symptoms and each index was subject to a square-root transformation to meet the assumption of homogeneity of variance (Table 4, Fig. 1).

**Table 3:** Mean number of symptoms and mean symptom distress indices by visit

	All Participants (*n* = 97)	
Index	Before starting ART, mean value [95% Cl)	After starting ART, mean value [95% Cl)
Total number of symptoms	12.8 (11.7–13.9)	7.8 (6.8–8.9)
Global Distress Index	1.29 (1.1–1.4)	0.7 (0.6–0.8)
Physical Symptom Distress	1.0 (0.9–1.1)	0.5 (0.4–0.6)
Psychological Symptom Distress	1.0 (0.8–1.2)	0.5 (0.4–0.6)

**Table 4:** Results for total number of symptoms and symptom distress indices when fitting linear mixed models to transformed sclaes

Parameter	Square root of total Number of Symptoms	Square root of Global Distress Score	Square root of Physical Symptom Distress Score	Square root of Psychological Symptom Distress Score
Constant	3.5 (3.4; 3.7)	1.1 (1.0; 1.1)	1 (0.9; 1.0)	0.8 (0.7; 0.9)
Days/100	−0.9 (−1.3; −0.6)	–	−0.4 (−0.5; −0.3)	–
(Days/100)^2^	0.2 (0.04; 0.3)	–	0.1 (0.02; 0.1)	–
√(Days/100)	–	−0.3 (−0.4; −0.2)	–	−0.3 (−0.4; −0.2)
σ_u_ (Between subjects)	0.5 (0.4: 0.7)	0.1 (0.06; 0.3)	0.1 (0.1; 0.2)	0.2 (0.2; 0.4)
σ_e_(Within subjects)	0.7 (0.6; 0.8)	0.4 (0.3; 0.4)	0.3 (0.3; 0.4)	0.5 (0.4; 0.5)

Note that each cell contains the parameter estimate together with 95% confidence limits where appropriate (i.e. if the given *parameter is* included in the model)

The model for square-root of the number of symptoms included terms in days post ART and days-squared, leading to the fitted equation for the predicted number of symptoms as follows: SYMPTOMfit = (0.164*(days/100)^2^–0.942*days/100 + 3.51)^2^.

The model for the square root of the Global Distress Index included a term for the square-root of the number of days post ART, leading to the following equation to predict the GDI as follows: GDIfit = (1.07–0.316 * √(days/100))^2^.

The model for the square root of the Physical Symptom Score included terms for day and day-squared, leading to the following equation: PHYSfit = (0.077*(days/100)^2^–0.402*days/100 + .951)^2^.

The model for the square root of the Psychological Symptom Score included a term for the square-root of the number of days post ART, leading to the following equation to predict the score as follows: PSYCHfit = (0.820–0.268 * √(days/100))^2^.

The fitted curve shows that the expected total number of symptoms declines from an initial value of 12.3 at day 0 down to 0.43 at 300 days. The decline is initially fairly rapid and then slows down (Fig. 1). The graph also shows the great variation between subjects; initially some individuals experience more than 25 symptoms, however after about 100 days no one has more than 20 symptoms and after about 200 days, no one has more than 10 symptoms.

The graph of observed and predicted GDI against days on ART shows that the expected GDI drops from an initial value of 1.15 to 0.27 by day 300 (Fig. 1). The initial drop in total number of symptoms is relatively quick and it then levels off. The graph of physical symptom scores against days on ART shows that the expected physical symptom score drops from an initial value of 0.90 to 0.19 by day 300. There is however a great deal of variability in this score and some participants still have scores close to 1 towards day 300. As the number of days on ART increases, the psychological symptom expected score drops from an initial value of 0.67 to 0.13 by day 300.

There was no association found between total number of symptoms or the symptom distress indices and ART regimen, CRYPTOPRO treatment arm (fluconazole vs. placebo) or CD4 cell count.

## Discussion

This longitudinal study of symptom burden during the first year of ART treatment shows that while ART reduces symptom profile in HIV infected individuals, certain symptoms retain a high burden. Of particular concern is the high burden and high distress associated with the top 10 most prevalent symptoms despite commencing ART caused by pain (60%), itch (57%), skin changes (53%), numbness and tingling in hand and feet (52%) and hunger (48%), cough (46%), worry (45%), lack of energy (43%), problems sexual activity (42%) and fatigue (41%).

In accordance with other studies we found that the greatest improvement in patient self-reported outcomes were in the initial months of ART with more modest reductions after longer periods of therapy [13, 14]. The MSAS-SF allows collection of extensive information on common physical and psychological symptoms, giving it the advantage of being a clinically useful tool. Symptom burden and quality of life instruments that group markers of wellbeing together enables the comparison of study participants with other populations with chronic illness and further, allows quantification of the outcomes of interventions [10]. While quality of life scores are an important outcome, global indices do not allow understanding of changes among individual symptoms. Distressing symptoms may be masked by improvements in global parameters.

There are notable improvements in the overall burden of the majority of both prevalent physical and psychological symptoms, but persistent or residual burden remains in some areas. About 60% of the participants continued to report pain, with one in five people with this symptom continuing to experience high distress. The causes of pain in the setting of HIV illness are multifactorial and it is particularly concerning in a setting with a lack of symptom control management and access to opioid analgesics. Numbness/tingling are reported in about half of study participants before and after commencing ART. Neuropathy is a common complication of HIV and is associated with advanced immunosuppression and low CD4 cell counts [20] and has been used as a predictor of death in resource limited settings [21]. In this study about 20% of patients developed a new symptom of peripheral neuropathy on commencing ART, but this was not related to the use of stavudine, a drug associated with neuropathy [22]. It is feasible that symptoms of pain and neuropathy could be managed effectively and at low cost with analgesics. It is of great concern that there is so little infrastructure available to address the profound symptom burden need [23].

Hunger does not decrease with commencing ART. In our study population, barriers to eating such as lack of appetite, nausea, a dry mouth and mouth sores all declined with ART and self-reported weight loss was reduced. However, hunger remained common and reported by about one-half of our participants throughout our study duration. A recent study conducted in Tanzania, Uganda and Zambia identified ART related hunger or not having enough food as a significant contributor to poor ART adherence [24]. ART driving increased appetite has been reported and food insecurity in poor populations cannot be overlooked. The integration of food assistance into HIV care programmes improves drug adherence [25].

ART was associated with clinically meaningful improvement in psychological symptom burden and feelings of being sad, irritable, and nervous and agreement with the statement “I do not like myself”. However, the psychological burden of worry remained in about a half of adults questioned and was a new symptom in about a third of patients commencing ART. The reasons behind these reports of psychological symptom burden were outside the scope of this study but one could postulate that, as the HIV infection moves from an acute to a chronic illness, a patient’s attitude changes as they realize that they are no longer at risk of an imminent death. Concerns about the future and pressures to adhere to ART, however, may explain the stubborn persistence of worry. A recent study of HIV infected individuals’ views on HIV treatment reported many negative psychosocial effects of ART, including a perceived trade-off between longevity and quality of life with issues surrounding career, finances, relationships with health care workers, housing security, sexual health and stigma [26].

The number of symptoms and symptom distress indices reduced to around half of their original score during the duration of this study. Improvements in quality of life subscales, but persistence of a relatively high frequency of specific symptoms after commencing ART is reported in other studies in sub-Saharan Africa [13–15]. Pitt and colleagues reported that despite a statistically significant improvement in health quality overall, the physical health score declined for nearly one quarter of the subjects and the mental health score declined for over one third [13]. In another study, the health related quality of life score for the study population after 12 months of ART had significantly improved, but nearly one-third of patients still experienced some or severe pain after 12 months of ART [15]. In a study of the impact of the first 6 months of ART on economic activity in South Africa [27] ART was associated with less functional impairment, fewer symptoms and better work performance. But over one-third continued to have functional impairment and the frequency of absenteeism, prevalence of body pain or headaches, fatigue, feeling unwell and depression all remained high. The reasons behind the lack of further improvement with ART are unclear but warrant further investigation; it may represent residual HIV symptoms, treatment related toxicity or complex psychosocial stresses. Data on long-term ART with immunological recovery from Western cohorts suggests a changing profile but persistent burden of symptoms [28–30]. The implication of this is that ART alone is not sufficient to reverse HIV-associated symptom burden and that clinical support to address physical and psychological needs will be required during the entire disease course.

This study highlights the significant impact that ART can have on improving symptom outcomes during the first year of initiation, but the enduring physical and psychological burden that remains is sobering. Recognition of a residual, often high, burden of symptoms is of key importance for clinicians and policy makers. While relief from pain and suffering is a basic human right [31] and requires no further justification, there are other reasons to pay attention to and monitor symptoms. Symptoms may well have a negative impact on ART adherence and risky behavior and hence HIV transmission. A study reported psychological symptom burden was significantly and independently associated with having unprotected sexual intercourse, and poor ART drug adherence [11]. Furthermore, physical symptoms including nausea and pain are known to be barriers to ART adherence [7, 32–34].

We believe that this study could have underrepresented the burden of symptoms in the population. Recruitment through a clinical trial with a defined inclusion and exclusion criteria means that participants may have differed in some ways from other HIV-infected Ugandans. The study participants were well enough to travel to a health clinic to be interviewed, and fulfilled strict trial criteria for enrolment. Further, our participants had access to trained medical staff, free diagnostics and treatment throughout the duration of the study and standard protocols existed for investigation and treatment of common HIV related conditions. More women were recruited than men, but the higher female to male ratio is consistent with the gender ratio starting HIV treatment in Uganda [35]. Although this was a trial population, the immune status of the study population was broadly representative of individuals at initial presentation for ART in sub-Saharan African countries at the current time [36]. We employed a counselor to assist in completing the questionnaire, although the counselors were trained this may have potentially introduced bias.

## Conclusions

While reductions in symptom burden indices following commencement of ART are encouraging, a high residual frequency of symptoms remains. Commencing ART heralds the mark of personal acceptance of HIV status. ART potentially allows individuals to return to work and normality, and contribute to society. However, the transition from a fatal illness to a chronic disease is placed at risk if symptoms are perceived to out-weigh benefits. On-going symptoms may erode previous improvements in behavior that reduce HIV transmission if symptom burden leads to poor ART adherence and attrition from HIV care [37]. It is essential that symptom control measures are applied through the disease and treatment trajectory and is valued for its practical and humanitarian gains.

## Additional file

Additional file 1: MSAS-SF questionnaire in English and Luganda available in the Additional file section. (DOC 85 kb)

## Abbreviations

ART: Anti-retroviral therapy
GDI: Global distress index
HIV: Human immunodeficiency virus
MSAS-SF: Memorial Symptom Assessment Scale-Short Form
PHYS: Physical Symptom Distress Score
PSYCH: Psychological Symptom Distress Score

## References

[1] Global AIDS Update 2016. [http://www.unaids.org/sites/default/files/media_asset/global-AIDS-update-2016_en.pdf]. Accessed July 2017.

[2] Selwyn PA, Rivard M, Kappell D, Goeren B, LaFosse H, Schwartz C, Caraballo R, Luciano D, Post LF (2003). Palliative care for AIDS at a large urban teaching hospital: program description and preliminary outcomes. J Palliat Med.

[3] Harding R, Molloy T, Easterbrook P, Frame K, Higginson IJ (2006). Is antiretroviral therapy associated with symptom prevalence and burden?. Int J STD AIDS.

[4] Wakeham K, Harding R, Bamukama-Namakoola D, Levin J, Kissa J, Parkes-Ratanshi R, Muzaaya G, Grosskurth H, Lalloo DG (2010). Symptom burden in HIV-infected adults at time of HIV diagnosis in rural Uganda. J Palliat Med.

[5] Harding R, Simms V, Penfold S, Downing J, Namisango E, Powell RA, Mwangi-Powell F, Moreland S, Gikaara N, Atieno M (2014). Quality of life and wellbeing among HIV outpatients in East Africa: a multicentre observational study. BMC Infect Dis.

[6] Harding R, Karus D, Easterbrook P, Raveis VH, Higginson IJ, Marconi K (2005). Does palliative care improve outcomes for patients with HIV/AIDS? A systematic review of the evidence. Sex Transm Infect.

[7] Sherr L, Lampe F, Norwood S, Leake Date H, Harding R, Johnson M, Edwards S, Fisher M, Arthur G, Zetler S (2008). Adherence to antiretroviral treatment in patients with HIV in the UK: a study of complexity. AIDS Care.

[8] Harding R, Clucas C, Lampe FC, Norwood S, Leake Date H, Fisher M, Johnson M, Edwards S, Anderson J, Sherr L (2012). Behavioral surveillance study: sexual risk taking behaviour in UK HIV outpatient attendees. AIDS Behav.

[9] Lampe FC, Harding R, Smith CJ, Phillips AN, Johnson M, Sherr L (2010). Physical and psychological symptoms and risk of virologic rebound among patients with virologic suppression on antiretroviral therapy. J Acquir Immune Defic Syndr.

[10] Harding R, Clucas C, Lampe FC, Date HL, Fisher M, Johnson M, Edwards S, Anderson J, Sherr L (2012). What factors are associated with patient self-reported health status among HIV outpatients? A multi-Centre UK study of biomedical and psychosocial factors. AIDS Care.

[11] Harding R, Lampe FC, Norwood S, Date HL, Clucas C, Fisher M, Johnson M, Edwards S, Anderson J, Sherr L (2010). Symptoms are highly prevalent among HIV outpatients and associated with poor adherence and unprotected sexual intercourse. Sex Transm Infect.

[12] Lowther K, Selman L, Harding R, Higginson IJ (2014). Experience of persistent psychological symptoms and perceived stigma among people with HIV on antiretroviral therapy (ART): a systematic review. Int J Nurs Stud.

[13] Pitt J, Myer L, Wood R (2009). Quality of life and the impact of drug toxicities in a South African community-based antiretroviral programme. J Int AIDS Soc.

[14] Stangl AL, Wamai N, Mermin J, Awor AC, Bunnell RE (2007). Trends and predictors of quality of life among HIV-infected adults taking highly active antiretroviral therapy in rural Uganda. AIDS Care.

[15] Jelsma J, Maclean E, Hughes J, Tinise X, Darder M (2005). An investigation into the health-related quality of life of individuals living with HIV who are receiving HAART. AIDS Care.

[16] Parkes-Ratanshi R, Wakeham K, Levin J, Namusoke D, Whitworth J, Coutinho A, Mugisha NK, Grosskurth H, Kamali A, Lalloo DG (2011). Primary prophylaxis of cryptococcal disease with fluconazole in HIV-positive Ugandan adults: a double-blind, randomised, placebo-controlled trial. Lancet Infect Dis.

[17] Chang VT, Hwang SS, Feuerman M, Kasimis BS, Thaler HT (2000). The memorial symptom assessment scale short form (MSAS-SF). Cancer.

[18] Finucane MM, Samet JH, Horton NJ (2007). Translational methods in biostatistics: linear mixed effect regression models of alcohol consumption and HIV disease progression over time. Epidemiol Perspect Innov.

[19] Rabe-Hesketh S, Skrondal A (2008). Classical latent variable models for medical research. Stat Methods Med Res.

[20] Schutz SG, Robinson-Papp J (2013). HIV-related neuropathy: current perspectives. Hiv/Aids.

[21] Birbeck GL, Kvalsund MP, Byers PA, Bradbury R, Mang'ombe C, Organek N, Kaile T, Sinyama AM, Sinyangwe SS, Malama K (2011). Neuropsychiatric and socioeconomic status impact antiretroviral adherence and mortality in rural Zambia. Am J Trop Med Hyg.

[22] Lichtenstein KA, Armon C, Baron A, Moorman AC, Wood KC, Holmberg SD, Investigators HIVOS (2005). Modification of the incidence of drug-associated symmetrical peripheral neuropathy by host and disease factors in the HIV outpatient study cohort. Clin Infect Dis.

[23] WHO global atlas on palliative care at the end of life. [http://www.thewhpca.org/resources/global-atlas-on-end-of-life-care]. Accessed July 2017.

[24] Koole O, Denison JA, Menten J, Tsui S, Wabwire-Mangen F, Kwesigabo G, Mulenga M, Auld A, Agolory S, Mukadi YD, van Praag E, Torpey K, Williams S, Kaplan J, Zee A, Bangsberg DR, Colebunders R. Reasons for missing antiretroviral therapy: results from a multi-country study in Tanzania, Uganda, and Zambia. PLoS One. 11(1):e0147309. doi:10.1371/journal.pone.0147309. 10.1371/journal.pone.0147309 PMC4720476 26788919

[25] Singer AW, Weiser SD, McCoy SI (2015). Does food insecurity undermine adherence to antiretroviral therapy? A systematic review. AIDS Behav.

[26] Park-Wyllie LY, Strike CS, Antoniou T, Bayoumi AM (2007). Adverse quality of life consequences of antiretroviral medications. AIDS Care.

[27] Rosen S, Ketlhapile M, Sanne I, Desilva MB (2008). Differences in normal activities, job performance and symptom prevalence between patients not yet on antiretroviral therapy and patients initiating therapy in South Africa. AIDS.

[28] Johnson MO, Neilands TB (2007). Coping with HIV treatment side effects: conceptualization, measurement, and linkages. AIDS Behav.

[29] Burgoyne RW, Rourke SB, Behrens DM, Salit IE (2004). Long-term quality-of-life outcomes among adults living with HIV in the HAART era: the interplay of changes in clinical factors and symptom profile. AIDS Behav.

[30] Johnson MO, Stallworth T, Neilands TB (2003). The drugs or the disease? Causal attributions of symptoms held by HIV-positive adults on HAART. AIDS Behav.

[31] Somerville M, Cotler I, Eliadis FP (1992). Human rights and medicine: the relief of suffering. International human rights law: theory and practice.

[32] Shah B, Walshe L, Saple DG, Mehta SH, Ramnani JP, Kharkar RD, Bollinger RC, Gupta A (2007). Adherence to antiretroviral therapy and virologic suppression among HIV-infected persons receiving care in private clinics in Mumbai, India. Clin Infect Dis.

[33] Hardon AP, Akurut D, Comoro C, Ekezie C, Irunde HF, Gerrits T, Kglatwane J, Kinsman J, Kwasa R, Maridadi J (2007). Hunger, waiting time and transport costs: time to confront challenges to ART adherence in Africa. AIDS Care.

[34] Muyingo SK, Walker AS, Reid A, Munderi P, Gibb DM, Ssali F, Levin J, Katabira E, Gilks C, Todd J (2008). Patterns of individual and population-level adherence to antiretroviral therapy and risk factors for poor adherence in the first year of the DART trial in Uganda and Zimbabwe. J Acquir Immune Defic Syndr.

[35] Keiser O, Anastos K, Schechter M, Balestre E, Myer L, Boulle A, Bangsberg D, Toure H, Braitstein P, Sprinz E (2008). Antiretroviral therapy in resource-limited settings 1996 to 2006: patient characteristics, treatment regimens and monitoring in sub-Saharan Africa, Asia and Latin America. Tropical Med Int Health.

[36] Braitstein P, Brinkhof MW, Dabis F, Schechter M, Boulle A, Miotti P, Wood R, Laurent C, Sprinz E, Seyler C (2006). Mortality of HIV-1-infected patients in the first year of antiretroviral therapy: comparison between low-income and high-income countries. Lancet.

[37] Atuyambe L, Neema S, Otolok-Tanga E, Wamuyu-Maina G, Kasasa S, Wabwire-Mangen F (2008). The effects of enhanced access to antiretroviral therapy: a qualitative study of community perceptions in Kampala city, Uganda. Afr Health Sci.

