# Supplementary material for: The impact of antiretroviral therapy on symptom burden among HIV outpatients with low CD4 count in rural Uganda: nested longitudinal cohort study
Source: BMC Palliat Care. 2017 Jul 13;17:8. doi: 10.1186/s12904-017-0215-y (PMC5508714; doi:10.1186/s12904-017-0215-y)
Supplement: Additional file 1: — MSAS-SF questionnaire in English and Luganda available in the Additional file section. (DOC 85 kb) [file 12904_2017_215_MOESM1_ESM.doc]

CRYPTOCOCCAL TRIAL – Symptom Burden Questionnaire

Clinic

date CDATE

VISIT[VIS] Enrolment[1] 3rd Follow up [2] 5^th^ Follow up [3]

Name

DOB or Age DOB/AGE

SP Number SPNO

Sc/Trial No TN

**MSAS-SF**

**Physical Symptom Burden in the past week**

1. Symptoms present or absent during the PAST WEEK **(Yes = 1/No =2)**

2. If **YES** then code level of distress with a code number. If **No** then leave blank.

- **Not at all 1**
- **A little bit 2**
- **Somewhat 3**
- **Quite a bit 4**
- **Very much 5**

|  | Symptoms present or absent during the PAST WEEK | **Yes=1**  **No=2** | **2. If yes how much distress did it course (code 1 – 5)** |
| --- | --- | --- | --- |
| **1** | Difficulty concentrating [DCON]  [OBUZIBU MU KUGOBERERA] |  |  |
| **2** | Pain [PAIN]  [OBULUMI] |  |  |
| **3** | Lack of energy [LENGY]  [OKUBULWA AMAANYI] |  |  |
| **4** | Changes in skin [CSKIN]  [OKUKYUKAKYUKA KW’OLUSUSU] |  |  |
| **5** | Numbness/tingling in the hands/feet [NUMB]  [OKUSANYALALA] |  |  |
| **6** | Sweats [SWTS]  [ENTUUYO] |  |  |
| **7** | Hair loss [HARL]  [OKUTUKA ENVIRI] |  |  |
| **8** | Itching [ITCH]  [OKUSIYIBWA] |  |  |
| **9** | Dizziness [DIZZ]  [KAMUNGULUZE] |  |  |
| **10** | Feeling drowsy/tired [DROW]  [OBUKOWU] |  |  |
| **11** | Cough [COUG]  [OKUKOLOLA] |  |  |
| **12** | Shortness of breath [SOB]  [OBUZIBU MU KUSSA] |  |  |
| **13** | Nausea [NAUS]  [OKUSINDUKIRIRWA EMEEME] |  |  |
| **14** | Dry mouth [DRYM]  [OKUKALA AKAMWA] |  |  |

Clinic date Trial number

______________________________________________________________

|  | Symptoms present or absent during the PAST WEEK | **Yes = 1**  **No = 2** | **2. If yes how much distress did it course (code 1 – 5)** |
| --- | --- | --- | --- |
| **15** | Difficulty swallowing [DSWAL]  [OBUZIBU MU KUMIRA] |  |  |
| **16** | Mouth sores [MSOR]  [AMABWA MU KAMWA] |  |  |
| **17** | Vomiting [VOM]  [OKUSESEMA] |  |  |
| **18** | Weight loss [WLOS]  [OBUKOVVU] |  |  |
| **19** | Lack of appetite [LAPT]  OBUTAYAGALA KULYA] |  |  |
| **20** | Changes in the way food tastes [TAST]  [ENKYUKAKYUKA MU BUWOOMI BW’EBYOKULYA] |  |  |
| **21** | Feeling bloated [BLOAT]  [OKUZIMBA OLUBUTO] |  |  |
| **22** | Constipation [CONSP]  [OBUZIBU MU KUFULUMA] |  |  |
| **23** | Diarrhoea [DIARR]  [OKUDUKANA] |  |  |
| **24** | Difficulty sleeping  [SLEP]  [OBUZIBU MU KWEBAKA] |  |  |
| **25** | Problems with sexual interest/activity [SEXA]  [OBUTAYAGALA KWEGATA] |  |  |
| **26** | Problems Urinating [URIN]  [OBUZIBU MU KUFUYISA] |  |  |
| **27** | Swelling of arms or legs  [SWEL]  [OKUZIMBA EMIKONO OBA AMAGULU] |  |  |
| **28** | I do not like myself [LIKE]  [OKWEKYAWA] |  |  |
| **29** | Sores lumps on my private parts  [LUMP]  [OBUWUNDU/OBUSUNDO MU BITUNDU BYEKYAAMA] |  |  |
| **30** | Discharge from my private parts [DISP] [OKUBUNDULA] |  |  |
| **31** | Bad smell/odour form body  [SMEL]  [AKASU OKUVA MUBIRI] |  |  |
| **32** | Difficulty moving [MOVE]  [OBUZIBU MU KWEKYUSA] |  |  |
| **33** | Difficulty walking [WALK]  [OBUZIBU MU KUTAMBULA] |  |  |
| **34** | Muscle aches [ACHE]  [OBULUMI MU BINYWA] |  |  |
| **35** | Problems seeing well- poor vision [VISN]  [OBUTALABA BULUNGI] |  |  |
| **36** | Difficulty hearing well – poor hearing [HEAR]  [OBUZIBU MU KUWULIRA] |  |  |
| **37** | Hunger [HUNG]  [ENJALA] |  |  |

Date Trial number

______________________________________________________________

|  | **Any other symptoms during the past week?** | yes | **2. If yes how much distress did it course (code 1 – 5)** |
| --- | --- | --- | --- |
| **38** | 1. | 1 |  |
| **39** | 2. | 1 |  |
| **40** | 3. | 1 |  |

**Psychological Burden of Distress in the past week**

1. Symptoms present or absent during the PAST WEEK **(Yes = 1/No =2)**

2. If **YES** then code level of distress. If No then leave blank.

- **Rarely 1**
- **Occasionally 2**
- **Frequently 3**
- **Almost constantly 4**

| Symptoms in the PAST WEEK | **Yes=1**  **No=2** | **If yes how often, code 1-4 ►** |
| --- | --- | --- |
| Feeling sad [SAD]  [OBUNAKUWAVU] |  |  |
| Worrying [WORRY]  [OBWERARIKIRIVU] |  |  |
| Feeling irritable [IRRIT]  [OKWEKYAAWA] |  |  |
| Feeling nervous [NERV]  OKUWULIRA NGA OTIDDE |  |  |

**Medication in the past week**

| Medication in the PAST WEEK | **Yes=1**  **No=2** |
| --- | --- |
| **ARV’s [ARV]** |  |
| **Septrin [SEP]** |  |
| **Trial drug [TD]** |  |
| **TB medication [TBRX]** |  |
| **Local medicine (name) [HERB]** |  |
| **Other (name) [OTH1]** |  |
| **Other (name) [OTH2]** |  |
| **Other (name) [OTH3]** |  |

Signature of staff completing Staff Number SN Date

Version1.0 4.12.07 Form C.P.MSAS
